# Protein Degradation and Protection Observed in the Presence of Novel Wound Dressing Components

**DOI:** 10.3390/jfb2040338

**Published:** 2011-12-02

**Authors:** Laura K.S. Parnell

**Affiliations:** Precision Consulting, 6522 Harbor Mist, Missouri City, TX 77459, USA; E-Mail: l-parnell@earthlink.net; Tel.: +281-208-3037

**Keywords:** wound, healing, inflammation, matrix metalloproteinase, tumor necrosis factor alpha, growth factors, systems biology, natural materials

## Abstract

Chronic wounds typically have excessive levels of matrix metalloproteinases (MMPs) and proinflammatory cytokines that impair healing. Reducing these detrimental proteins may be key to healing chronic wounds. Proprietary protease blends were formulated specifically to degrade excessive amounts of proinflammatory factors that could prevent wound healing. Applications of protease-containing wound dressings to acute and chronic wounds have been observed clinically to resolve inflammation and appear to aid healing. The purpose of this study was to test *in vitro* a deliberate blend of proteases for the ability to deactivate or activate known proteins associated with inflammation or healing. Purified human target proteins were incubated with test and control solutions and samples removed at various time points. Blinded samples were tested using a novel infrared protein multiplex sandwich-ELISA-type array technique. Many proinflammatory proteins such as MMPs, cytokines and chemokines were susceptible to degradation. Many proteins such as growth factors, cytokines and TIMP1 were resistant to degradation. Not all proinflammatory proteins were deactivated. Family protein structure did not appear to affect susceptibility to degradation or deactivation. These results suggest that specific protease containing wound dressings appear to reduce multiple detrimental components which may disrupt their deleterious effects on the wound bed and microenvironment. By improving the wound microenvironment through the use of definitive proteases, these novel wound dressings may help transition wounds into the subsequent phase of healing.

## 1. Introduction

Current understanding of the physiology and immunology of wound healing has evolved from Winter's observations of a moist wound environment, to a better understanding of the complexities of the wound microenvironment [1,2,3]. The microenvironment of wounds, burns and scars is a complicated mix of interdependent signaling pathways, cells, proteins, enzymes and resulting interactions that regulate and direct tissue repair and remodeling [2,4,5,6,7]. Within these pathways, each cell has multiple receptors that are constantly regulated by endocrine, paracrine, and autocrine signals resulting in up or down-regulation of cell function and protein secretion further contributing to the network of signals (*i.e.*, cytokines, growth factors, hormones, *etc.*) [5,8,9]. Even in the most simplistic wound, the activities and functions of individual cellular and molecular items are intricately involved in the microenvironment [2,3,10,11].

In the normal healing process, redundant or backup signals allow healing to occur even if impairment exists in another signal pathway [2]. A balance between MMPs and TIMPs are tightly regulated as are the factors involved in the inflammatory and proliferative stages. Research on cytokines and transgenic knock-out mice has clearly shown that healing may be delayed by one dysfunctional component or gene, but very few components can prevent healing completely unless coupled with other dysfunctional components, genes, or pathways because of duplicate feedback loops [2,4,10,12,13,14,15]. Strength of redundant feedback loops is signal amplification which can be up-regulated or down-regulated within seconds because of the interactions between the signaling networks.

In nonhealing chronic wounds, however, excessive signal amplification can become a detrimental weakness. When an imbalance in the regulation between MMPs and TIMPs develops, the physiological response becomes skewed and will continue indefinitely until counterbalanced signals can move the response back to more normal levels. An imbalance can be caused by a variety of substances such as age, injury, micro-organisms and their secretions, immune cells and their secretions, cytokines, chemokines, inflammatory factors, dead and apoptotic cells, and free radicals. To further complicate the matter, MMPs can not only self up-regulate their presence in a wound, but also signal for additional inflammation via proinflammatory cytokines and chemokines. Furthermore, MMPs can destroy growth factors, cytokines and extracellular matrix, all of which could act as counterbalances if allowed to persist in the wound [16,17,18,19]. Additional environmental proteases (*i.e.*, neutrophil elastase) from self or micro-organisms can inactivate TIMPs allowing a surge in proMMP and active MMP activity levels [20]. This hostile microenvironment becomes a perpetual cycle of imbalance and the wound cannot close [16,17,18,19]. This imbalanced microenvironment has been clearly demonstrated in chronic wounds with excessive levels of MMPs and proinflammatory cytokines [16,17,18,19,21].

In order to try and interrupt the imbalanced chronic wound cycle and start the healing, methods of debridement, growth factor therapy and chelation have been tried [22,23,24]. Past use of individual molecules and compounds to modify the healing process at a specific point has been met with limited success possibly because of imbalanced and redundant networks [23,25,26]. Debridement and preparing the wound bed to convert a chronic wound to a more acute type wound microenvironment is widely accepted [22].

Based on the interdependencies of cells and signaling pathways as well as the pathophysiology of wounds, we began developing wound treatments that could impact multiple signaling factors. Endopeptidases can act as catalysts to cleave specific internal peptide bonds in proteins and were a logical choice. The proteases in this project were formulated specifically to deactivate excessive amounts of proinflammatory factors that could prevent wound healing. An effort to choose proteases to conserve potentially beneficial proteins such as growth factors was made. By reducing multiple nocuous factors, various autocrine and paracrine feedback loops could be down-regulated or disrupted. As a result, shifting the imbalanced wound microenvironment toward more balanced signal cross-talk could theoretically transition the wound into the next phase of healing. The protease blends have since been developed into wound and skin dressings (Protease Technology^®^, Swiss-American Products, Carrollton, TX, USA).

The purpose of this study was to further elucidate the intensity and speed of particular proteases affecting key wound healing proteins. The blend of protease enzymes used in the dressings was tested for the ability to degrade, deactivate or activate known inflammatory or healing proteins. Some test results using a variety of methods and proteins were previously published [27]. Additional and repeat proteins using the same blend have been tested with a new technique to corroborate prior findings and are published herein.

## 2. Experimental Section

### 2.1. Sample Preparation

The protease blend currently used in wound and skin dressings was tested as a solution. Control and test solutions (Swiss-American Products, Carrollton, TX, USA) were provided to an independent lab (MicroConsult, Dallas, TX, USA). Purified human target proteins (Aushon Biosystems, Billerica, MA, USA) associated with inflammation and/or wound healing were selected for testing and shipped to the lab (MicroConsult, Dallas, TX, USA). At the lab, human target proteins were reconstituted with PBS per manufacturer's directions and incubated at 37 °C with control PBS (without protease blend) and test PBS (with protease blend) solutions. The pH was tested to confirm pH levels were within 0.05 of one another. No samples required pH adjustment. Timed samples were taken at 0, 1, 4, 8, and 24 h and mixed 100:1 with a general purpose protease inhibitor to stop any enzymatic reactions including those involving MMPs (P2714, Sigma; St. Louis, MO, USA). Samples were blinded and aliquoted prior to freezing at −80 °C.

### 2.2. Sample Testing

Blinded samples were placed on dry ice and shipped overnight for testing with an infrared protein multiplex sandwich-ELISA-type array technique (SearchLight Sample Testing Service of Aushon Biosystems, Billerica, MA, USA). The Aushon testing service is a CLIA (Clinical Laboratory Improvement Amendments) certified laboratory that follows strict GLP (Good Laboratory Practices) regulations, procedures and protocols as required by the US Government.

The SearchLight Technology testing service creates and validates each specific protein antibody array based on the biomarkers selected for testing. Each custom array can measure up to 16 biomarkers at once that reduces the sample size required and can measure potential protein interactions within the samples if desired. The multiplex array uses an antibody sandwich-ELISA-type technique that is spot arranged within each well. Blinded samples were tested with internal SearchLight controls a minimum of three times and the average result and variance was provided in the final report. Approximately 50 target proteins associated with inflammation and healing were tested including MMPs, TIMP, cytokines, chemokines, receptors, neuropeptides and growth factors.

### 2.3. Statistical Methods

SearchLight data were analyzed using separate repeated measures analysis of variance model with time, treatment, and time-by-treatment as fixed effects in the model with a compound symmetry correlation structure. All statistical calculations were made using the software program Analyse-It^®^ (Analyse-It Software, Ltd., Leeds, UK).

## 3. Results and Discussion

Many proteins were tested, however, due to space constraints, only the most pertinent results are reported here.

### 3.1. Matrix Metalloproteinases (MMPs) and Tissue Inhibitor of Matrix Metalloproteinases (TIMPs)

**Figure 1:**
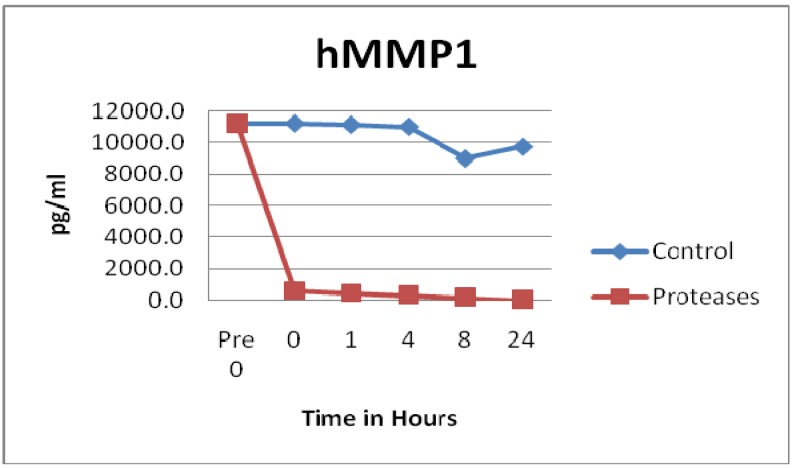
MMP1 observations showed rapid degradation by the tested protease blend (n = 48). Degradation was statistically significant (p = 0.0011).

**Figure 2:**
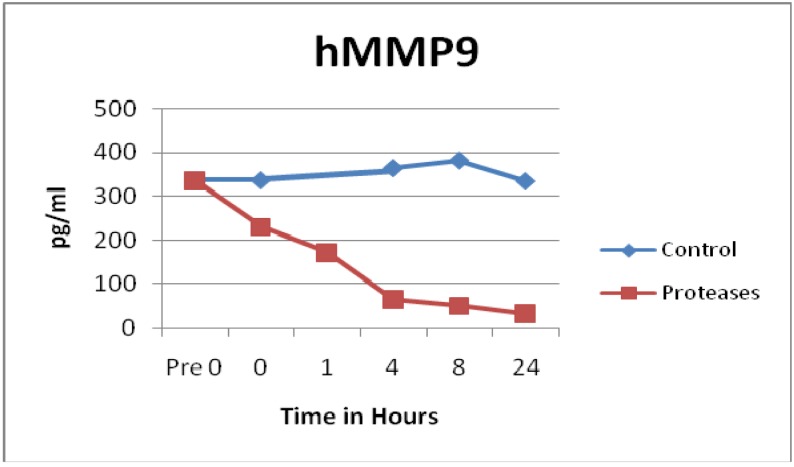
MMP9 observations showed rapid degradation by the tested protease blend. Degradation was statistically significant (n = 24, p = 0.0051).

Many proinflammatory enzymes such as matrix metalloproteinases (MMP) were degraded by the tested blend of proteases. Human MMP1 (p = 0.0011), hMMP2 (p = 0.0044), hMMP3 (p = 0.1187), hMMP7 (p = 0.0784), hMMP8 (p = 0.0258), and hMMP9 (p = 0.0051) all showed degradation of various degrees by the protease dressing blend. The most impressive degradation was for MMP1, which was degraded to near zero levels within seconds of adding the protease blend, see Figure 1. Also, MMP9 showed a rapid degradation within 4 h, see Figure 2. Prior ELISA assay results of the protease blend incubated with chronic wound fluid with known MMP9 levels, showed complete MMP9 degradation within one hour [27]. Even though both assays showed rapid degradation, at this time, it is not known why there is a slight difference of time of degradation observed.

**Figure 3:**
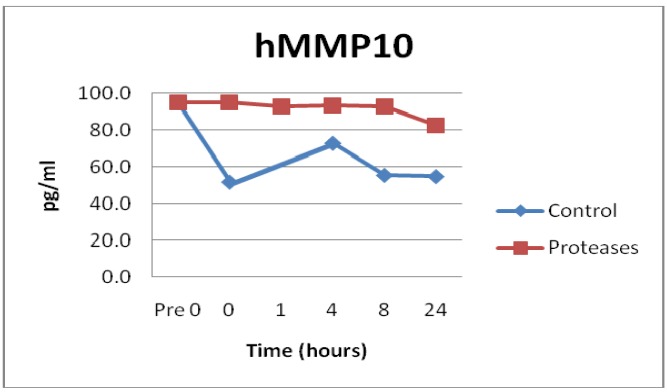
MMP10 observations showed relatively little degradation by the tested protease blend even after 24 h at 37 °C. Difference compared to control PBS solution degradation was statistically significant (n = 24, p = 0.0129).

**Figure 4:**
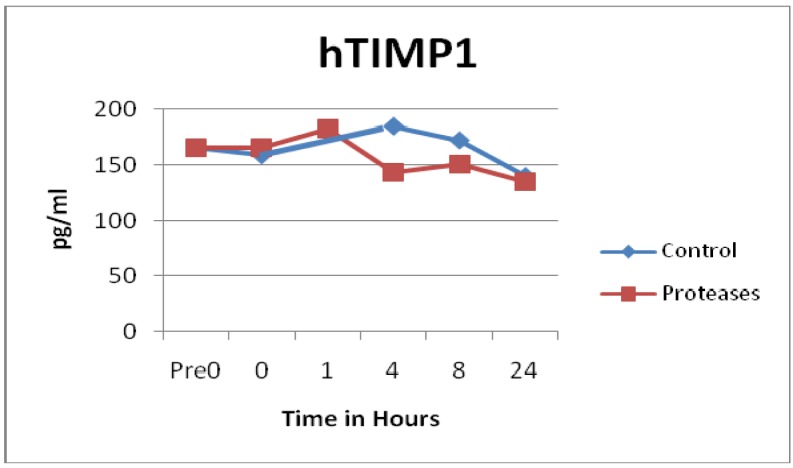
TIMP1 observations showed similar degradation over time between control and test solutions (n = 48, p = 0.5194).

Not all MMPs were degraded by the proteases. In fact, control solutions saw more degradation than solutions containing the proteases in the MMP10 (p = 0.0129) and MMP13 (p = 0.0186) samples, see Figure 3. Some of the MMP enzymes degraded within seconds, others took hours, and some had little to no degradation detected, see Figure 1, Figure 2 and Figure 3. Tissue inhibitor of matrix metalloproteinase 1 (TIMP-1) showed similar degradation over time for both the control and test samples and were not significantly different (p = 0.5194), see Figure 4. The SearchLight technique results using pure TIMP-1 showed little degradation and are very similar to results previously reported using chronic wound fluid with an ELISA assay [27].

### 3.2. Cytokines and Chemokines

Many proinflammatory cytokines and chemokines were degraded by the protease blend. Both tumor necrosis factor α (TNFα) active monomer (p = 0.0017) and TNFα active trimer (p = 0.0195) showed rapid degradation. The active monomer degraded to near zero levels within seconds whereas the active trimer degraded 75% within one hour of incubation with the protease blend. Previous results utilizing chronic wound fluid and purified TNFα (a variety of protein structures) showed rapid and complete degradation within 8–10 h [27].

**Figure 5:**
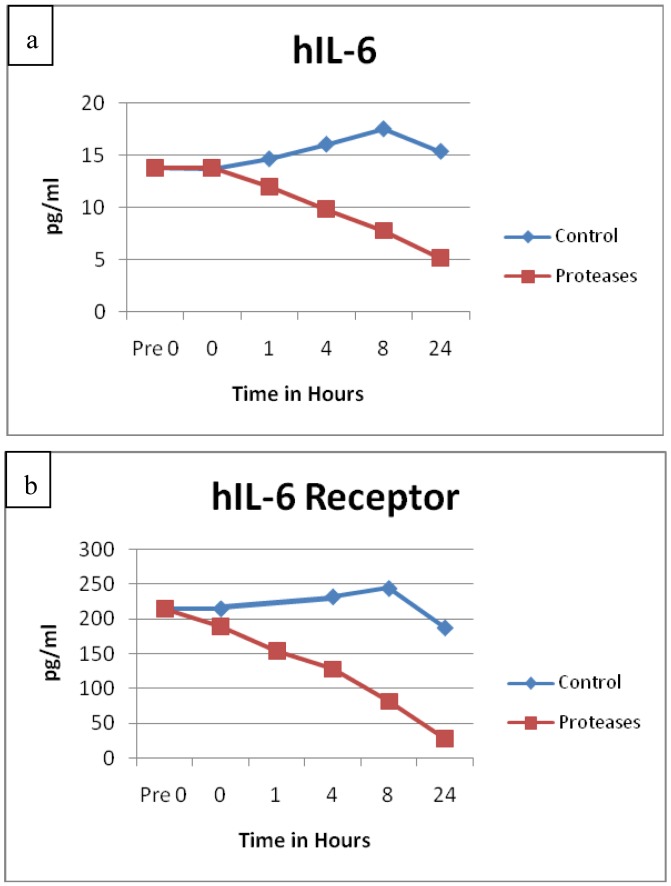
(**a**) IL-6 and (**b**) IL-6 receptor observations showed remarkable similarity in their degradation patterns over time. Degradation was statistically significant for both p = 0.0111 (n = 24) and p = 0.0260 (n = 24) respectively.

The protein degradation of Interleukin-6 (IL-6, p = 0.0111) and its receptor (IL-6R, p = 0.0260) had remarkably similar degradation patterns, see Figure 5(a,b). The protein structures for IL-6 and its receptor are very different, yet both exhibited sustained degradation over time in similar fashions. Significant degradation for IL-2 (p = 0.0048), granulocyte macrophage colony stimulating factor (GMCSF, p = 0.0017), macrophage inflammatory protein 1 α (MIP1α, p = 0.0207) and interferon α (IFNα, p = 0.0119) were also detected. All of these proteins are known to be involved in areas of inflammation. MIP1α is also known as chemokine (C-C motif) ligand 3 (CCL3).

**Figure 6:**
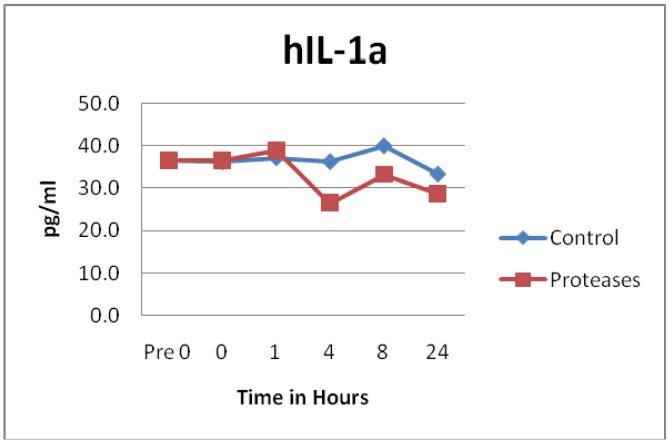
IL-1α observations showed similar degradation over time between control and test solutions (n = 24, p = 0.1633).

Other cytokines and chemokines did not appear to have any statistically significant differences between the control and protease solution degradation. These included IL-1α (p = 0.1633), IL-1β (p = 0.2124), IL-8 (p = 0.1027), IL-10 (p = 0.4224), IL-12p70 heterodimer (p = 0.0694), IL-18 (p = 0.1118), IL-23 (p = 0.3496), Interferon γ induced protein 10 (IP10, p = 0.1232), MIP1β (p = 0.7103) and macrophage chemotactic protein 1 (MCP1, p = 0.0868). See Figure 6 as an example of results typically seen. Previous incubation of chronic wound fluid with known IL-1β levels with dressing proteases and assayed by ELISA showed similar results as the SearchLight data reported here. It should be noted that IP10 is also known as C-X-C motif chemokine 10 (CXCL10) and MIP1β is also known as CCL4.

### 3.3. Growth Factors

**Figure 7:**
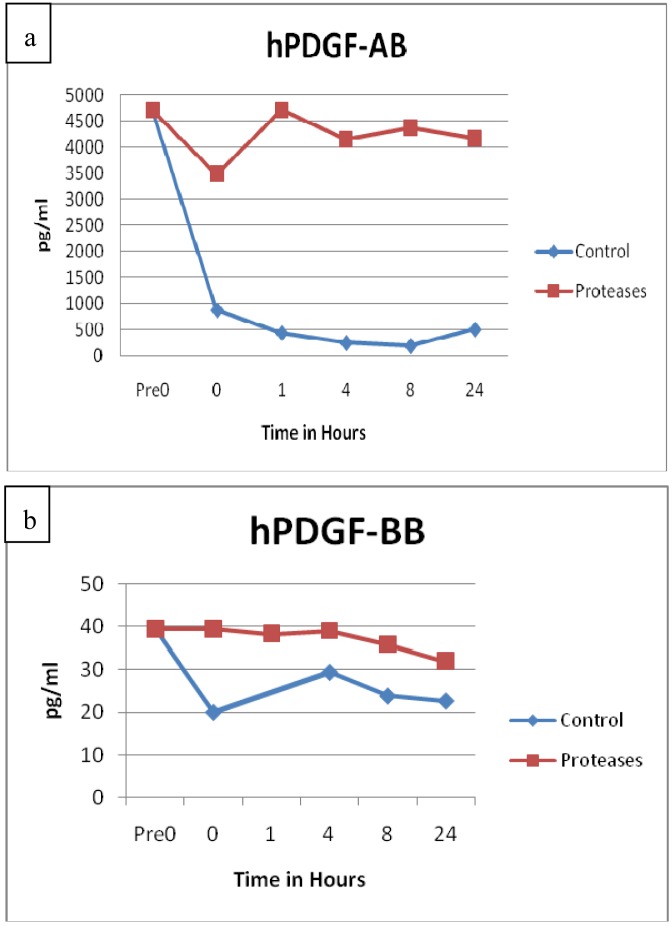
(**a**) PDGF-AB and (**b**) PDGF-BB observations showed statistically significant resistance to degradation over time in the protease solutions compared to control solutions (PDGF-AB, n = 24, p = 0.0002; PDGF-BB, n = 48, p = 0.0146).

Growth factors were also tested for degradation. Growth factors samples of vascular endothelial growth factor (VEGF, p = 0.2729), transforming growth factor β (TGFβ, p = 0.6075) and fibroblast growth factor basic (FGF basic, p = 0.4477) did not appear to have any statistically significant differences between the control and protease solution degradation. Both platelet derived growth factor AB (PDGF-AB, p = 0.0002) and PDGF-BB (p = 0.0146) maintained higher concentrations in the protease solutions throughout the 24 h period than the control solutions, see Figure 7(a,b). Prior ELISA assay of the proteases with PDGF-AB and chronic wound fluid also showed sustained growth factor levels throughout the 24 h testing period, whereas the controls showed degradation [27]. None of the growth factors incubated with the protease blend had any significant degradation.

### 3.4. Neuropeptides

**Figure 8:**
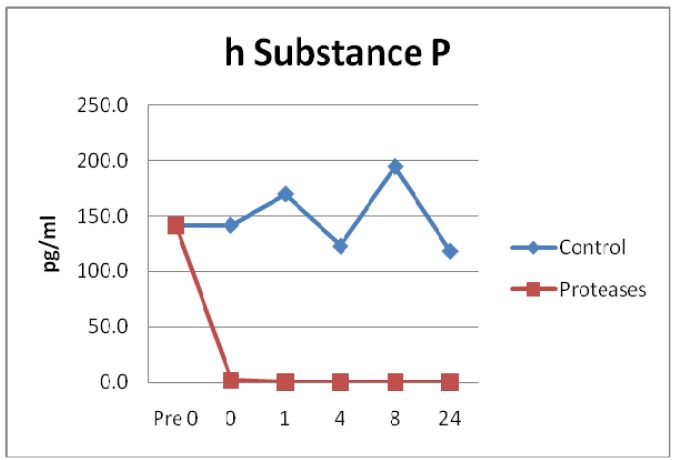
Substance P observations showed rapid degradation by the tested protease blend within seconds of exposure. Degradation was statistically significant (n = 24, p = 0.0008).

A neurotransmitter, Substance P (SP) and a neurotrophic protein Glial cell-derived neurotrophic factor (GDNF) were incubated with the protease solution and degradation measured. SP degradation in the protease solution was statistically significant compared to control (p = 0.0008). Degradation of SP occurred within seconds of the addition of the protease test solution, see Figure 8. Conversely, GDNF control and test groups did not have significant degradation (p = 0.8682).

### 3.5. Results Summary

Proteins MMP1, MMP2, MMP8, MMP9, TNFα active monomer, TNFα active trimer, IL-2, IL-6, IL-6R, GMCSF, MIP1α, IFNα and SP were susceptible to degradation by the protease solutions. Proteins TIMP1, IL-1α, IL-1β, MIP1β, VEGF, TGFβ, FGF basic and GDNF were resistant to degradation and showed levels similar or higher than control solutions up to 24 h of incubation. Throughout the 24 h incubation period, concentration levels of PDGF-AB and PDGF-BB were statistically higher in protease treated samples than the controls.

**Figure 9:**
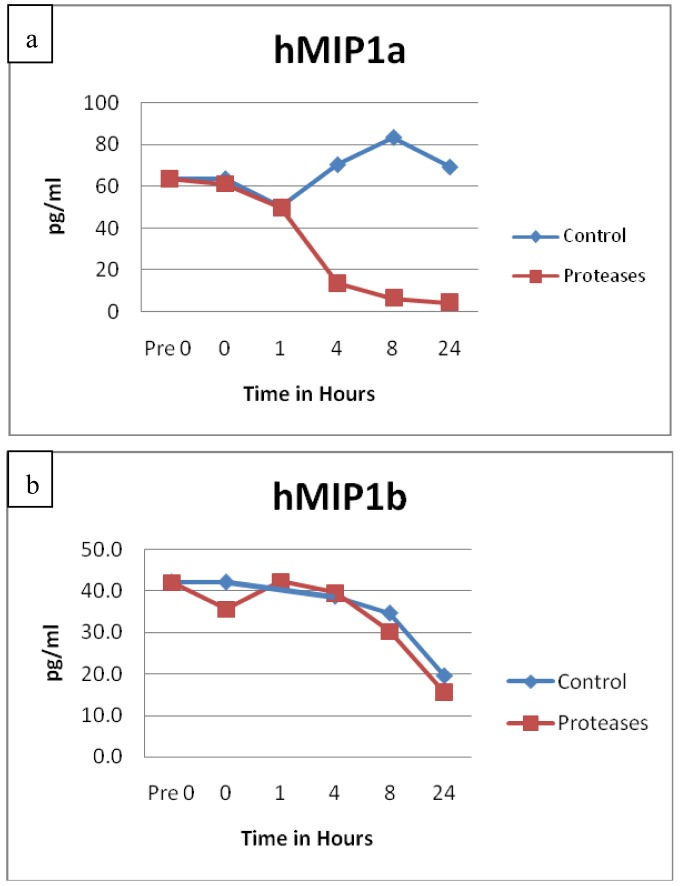
**(**a**)** MIP1α and (**b**) MIP1β observations show very different degradation patterns over time. Degradation was statistically significant for MIP1α (n = 24, p = 0.0207) but not MIP1β (n = 24, p = 0.7103).

Family protein structure did not appear to affect degradation or resistance, but instead was determined by individual protein structure. A good example of this can be seen in Figure 9(a,b) comparing MIP1α and MIP1β. Also, even though the protein structures are different, IL-6 and IL-6R demonstrated remarkably similar degradation patterns. Although the AB heterodimer and BB homodimer of PDGF are structurally different, both showed sustained concentrations over time in the presence of the protease blend.

The purified protein solutions used in this experiment produced similar results as collected wound fluids with known protein amounts [27]. The new SearchLight array technique showed consistent, reproducible results compared to standard ELISA technique used previously. Unlike the ELISA assay, the SearchLight technique was faster and required a smaller sample size.

### 3.6. Discussion

In chronic wounds, research has shown that detrimental components outnumber beneficial components in the wound milieu [16,17,18,19,21]. MMPs, elastases, collagenases, proinflammatory cytokines, chemokines and neuropeptides are known to be destructive to the healing process when produced in excess [2,18,24,28,29]. Not only do these components up-regulate themselves through feedback loops and perpetuate an imbalance, MMPs and other enzymes can destroy TIMPs, growth factors and tissue that normally would provide counterbalance via the same networks [16,17,18,19,20,30]. Such an imbalance will not lead to healing until the microenvironment becomes more balanced.

Unique protease properties were selected for specific targets of degradation and protection. Choosing distinct proteases allows wound dressings to be customized such that a formulation for phagocyte driven inflammation could be different than one for T cell driven inflammation. Applications of a specific blend of anti-inflammatory proteases to a chronic wound could decrease the level of numerous detrimental components. By using protease blends as immunomodulators, the imbalanced pathways and cross-talk should shift towards a more physiologically balanced microenvironment as multiple detrimental components are degraded. The physiologic effect should be broad and amplified because multiple specific proteins are targeted instead of one sole protein. The end result should lead to improved wound healing. The chosen proteases were formulated into wound and skin dressings.

The protease blend tested from wound and skin dressings showed degradation of specific proteins, receptors, and enzymes that in excess impair healing by promoting inflammation. In this experiment and previously demonstrated using spiked chronic wound fluid, excess proinflammatory mediators were degraded by the protease blend, but excess growth factors were not [27]. It is important to note that neutrophil elastase is known to degrade PDGF, TGFβ and TIMPs whereas the formulated proteases do not [20,27,29]. Unlike some wound dressings, these study results showed many proteins beneficial to healing and angiogenesis were not degraded. Not all proinflammatory mediators were deactivated suggesting the protease blend would not eliminate the inflammatory response but, instead, could reduce the intensity. Elimination of the inflammatory response or complete removal of an active protein (*i.e.*, MMPs) is not desirable because the microenvironment imbalance could shift from one extreme to the other and still fail to achieve closure. Thus, it is more important to dampen the signal of multiple key proteins than to eliminate or add one factor involved in tissue repair.

Endopeptidase enzymes are present in human, animal, and plant tissues. The proteases used in this project were screened and are generally well tolerated by the human body. Formulated proteases applied directly to the dermal wound bed have not elicited any complaints or adverse events [31,32,33]. Because many proteases are potent catalysts, low concentrations can deactivate specific components. Low concentrations are beneficial for two reasons. The less exogenous proteins applied to a wound (1) the less likely irritancy will occur, and (2) the body can self-eliminate the proteins. Self-elimination of the proteases also allows the regulation pathways to proceed without shifting too far in the opposite pathway direction (*i.e.*, excessive collagen synthesis). Use of certain extrinsic proteases to impact the regulatory healing processes represents a simplistic, yet novel approach to improving wound healing. This technology utilizes the strength of the feedback loops in order to modulate the wound.

Topical wound and skin dressing products have been formulated using the protease blend reported here. Dramatic improvements in inflammation are consistently seen within 12–48 h following application of protease containing wound and skin dressings [31,32,33,34]. Clinical symptoms of inflammation such as itch and heat often improve or resolve quickly in minutes whereas erythema and edema typically take longer depending on the wound. In chronic wounds, the fourth day following consecutive protease containing dressings often show wound beds that are beginning to differentiate and re-epithelialize [32]. These improved clinical observations have been documented in chronic contact dermatitis, acute and chronic fungal perineal dermatitis, chronic pressure ulcers and burn scar itch [31,32,33,34]. These etiologies all clinically display inflammation and associated symptoms and are known to have elevated levels of proinflammatory mediators (*i.e.*, TNFα, MMPs, *etc.*).

**Figure 10:**
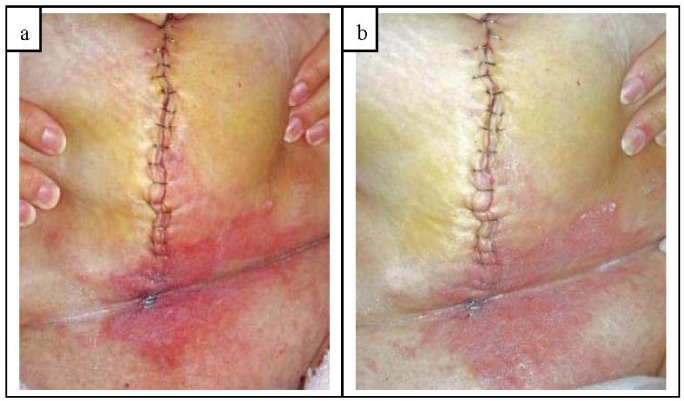
(**a**) At post-op day 4, patient complained of pain across lower belly and pannus with intense itching across lower half of incision and pannus. Area was red, irritated, warm, moist and tender to the touch exhibiting classic symptoms of fungal/yeast infection. Area was cleaned and an antifungal dressing containing the protease blend applied; (**b**) Clinical improvement within 11.5 h following protease containing antifungal application was seen. Patient comfort improved and had significant decrease in itch, pain, inflammation, erythema, warmth, irritation and edema. Note the vast improvement in skin color, texture and irritation. Entire infected area fully resolved within 48 h.

Here is a clinical example showing rapid improvement in inflammation when a protease blend containing dressing was used. A patient underwent a radical hysterectomy with bilateral salpingo-oophorectomy and lymph node resection. At post-op day 4, patient complained of pain across lower belly and pannus with intense itching across lower half of incision and pannus. Examination revealed inflammation, vesicles and purulent exudate from base of incision up to and including the umbilical area. The lower pannus, abdominal fold and pubic area was red, irritated, warm, moist and tender to the touch exhibiting classic symptoms of fungal/yeast skin infection, see Figure 10(a). The incision line and the infected skin was cleansed and patted dry. A 2% miconazole antifungal cream with a protease blend (Trivase^®^, Swiss-American Products, Carrollton, TX, USA) was applied to all inflamed areas with special attention to areas of itching. The incision line was dressed with an alginate dressing and secured with a secondary dressing. All systemic medications remained unchanged. Within 11.5 h following the Trivase application, the patient had significant improvement in pain and itch across the lower belly, pannus and pubic area. An examination revealed a marked decrease in inflammation, redness, irritation, and warmth. The degree of improvement in erythema and inflammation can be readily seen in Figure 10(b). Tenderness, pain and itch were improved such that subsequent cleansing and reapplication of Trivase were easy with little discomfort. The entire fungal infected area under the pannus fully resolved within 48 h.

Obviously patient improvement depends on the individual factors involved, but compared to patients with similar symptoms and history, the improvement was markedly rapid. Normally a similar patient treated with a 2% miconazole cream without the protease blend would be expected to have appreciable symptomatic relief in one to two days and resolution of the infection in four to five days. However, the protease blended 2% miconazole cream used here had symptomatic and visual improvement within 12 h. The skin infection was completely resolved within 2 days whereas traditional miconazole antifungal creams would require several additional days of treatment. Such an improvement in patient comfort, morbidity and decrease in time to closure helps lower the risk of additional secondary infection and improves patient outcomes.

The clinical findings and *in vitro* study results appear to correlate well, especially in regards to the rapid reduction in MMPs, TNFα and SP with the rapid resolution of inflammatory symptoms. In wounds and skin displaying inflammation, an application of protease containing dressings typically improves patient comfort as inflammation, specifically symptoms of pain, itch and heat, resolve. As the inflammation resolves, the wound and tissues become healthier looking with less erythema and edema resulting in a firmer, less painful, healthy wound bed. An improvement in patient quality of life and a reduction in analgesic medication are expected to be significant and are being studied.

Thus far, the proteases have been stabilized and used within vehicles of hydrogel, ointment, creams and lotion for use on compromised and intact skin as well as acute and chronic wounds. The vehicle used affects the release rate speed for quick action or sustained exposure. Incorporating the protease formulation into non-amorphous dressings and scaffolds are being investigated and new formulations and compounds are being developed. Protease containing wound dressings need to be in contact with the wound bed for optimal release and effect. To avoid frequent dressing changes, the dressings need to be engineered such that the protease concentration is maintained throughout the wear time. Use of a protease coated degradable scaffold (*i.e.*, collagen and hyaluronic acid) for tissue repair is exciting because the inflammatory cycle could be held in check and the materials needed for tissue repair would be readily accessible to the body. Clinical problems of adhesion formation following abdominal or gynecological surgery might be avoided or minimized by use of a protease containing gel or film. In acute procedures such as laser resurfacing, inflammation, wound bed protection and cosmesis are important factors the first 48–72 h. A protease containing wound dressing that is applied as a solution that forms a protective film would be desirable to both surgeons and patients. Combining customized protease formulations into various biomaterials could eventually lead to more effective wound healing for a variety of wounded tissues.

It should be noted that these proteases and wound dressings differ greatly from enzymatic wound debriders. Commercial enzymatic debriding agents contain high concentrations of specific or non-specific proteinases, with or without hydrolases in an ointment base [35,36]. Enzymatic debriders are intended to debride slough and wound eschar components in order to prepare the wound bed by removing devitalized tissue. In contrast, the protease wound dressing formulations are designed to impact specific cytokines, proteins and proteases, not devitalized tissue. Although all debriding agents work towards physical removal of nonviable macroenvironment tissue, not all wound debriders can preserve viable tissue and growth factors [36]. Conversely, the proteases in this project are selective for specific microenvironment proteins which are found near but not in devitalized tissue. As a result, the protease blend ingredients are different and overall units of activity are substantially lower than commercially available enzymatic debriders.

The SearchLight microarray technique used was rapid, inexpensive, and required small sample sizes. Overall, the technique showed reproducible results when compared with previously assayed ELISA results. This technique is an excellent screening tool although it may have slightly more variability than comparative ELISA assays. However, since multiple proteins can be tested at the same time, it conserves both time and expense. Proteins can be run separately or together making it possible to observe protein interactions during incubation without requiring additional steps prior to assaying.

Like ELISA assays, the SearchLight microarray technique measures the antibody capture of the target which may or may not be similar to biological activity levels. Use of validated cell or protein based bioassays will be necessary to correlate the biological activity with the results published herein. Some initial testing suggests certain biologically inactive pro-forms of proteins can be cleaved and activated following incubation with the proteases at various concentrations, but the preliminary results are inconclusive (unpublished data). It is also possible that during the protease incubation period, the tertiary or quaternary folded structure could be opened allowing high levels of the target to be detected via the microassay, but remain biologically inert. Testing samples with the SearchLight microassays and bioassays should help determine the extent of protein activation/deactivation during incubation with the proteases.

The SearchLight technique can accommodate a variety of animal and human fluids, tissue homogenates and culture media samples; therefore, future experiments should be able to measure subject sample changes in conjunction with the clinical observations. Many of the inflammatory factors are highly conserved in mammals and it is theorized the protease blend would behave similarly in animal wounds. A porcine wound healing model with protease containing and control wound dressings could allow tissue samples to be taken during the healing process. Samples analyzed with SearchLight microarray could then be compared to the wound characteristics and healing outcomes. A double-blinded, randomized controlled trial (RCT) pilot study evaluated the use of a protease containing lotion to relieve pruritus in post burn itch subjects with encouraging results [34]. Control and test materials were applied to intact skin, but no tissue sampling was performed due to ethical reasons. Additional RCTs are being discussed in the areas of post burn itch and psoriasis. Developing validated fluid sampling techniques appear to be essential in order to evaluate clinical outcomes with dynamic microenvironmental changes in open and compromised skin. Clinical research with protease containing products to further corroborate the *in vitro* findings is ongoing.

## 4. Conclusions

In the chronic wound milieu, excessive levels of MMPs, elastases, collagenases, proinflammatory cytokines, chemokines and neuropeptides are known to impair healing. Using a blend of proteases to lower the availability of multiple nocuous proteins appears to help the microenvironment become more balanced. Documentation of rapid resolution of clinical inflammatory symptoms and the quick reduction in pro-inflammatory proteins in the *in vitro* studies suggest high correlation between the two. Use of definitive proteases in novel wound dressings may improve the wound microenvironment such that healing can proceed.
